# Safety, tolerability and efficacy of intra-articular Progenza in knee osteoarthritis: a randomized double-blind placebo-controlled single ascending dose study

**DOI:** 10.1186/s12967-018-1420-z

**Published:** 2018-03-06

**Authors:** D. Kuah, S. Sivell, T. Longworth, K. James, A. Guermazi, F. Cicuttini, Y. Wang, S. Craig, G. Comin, D. Robinson, J. Wilson

**Affiliations:** 1 Sydney Sportsmed Specialists, Park House, Level 3, 187 Macquarie Street, Sydney, NSW 2000 Australia; 2 Regeneus Ltd, Ground Floor, 25 Bridge St, Pymble, NSW 2073 Australia; 3 Sydney Sports Medicine Centre, Level 2, NSWIS Building, 6 Figtree Drive, Sydney Olympic Park, NSW 2127 Australia; 4 FNQ Sports Medicine, 225-227 Draper Street, Cairns, QLD 4870 Australia; 5 Quantitative Imaging Center, Department of Radiology, Boston University School of Medicine, Boston, MA 02118 USA; 6 Department of Epidemiology and Preventative Medicine, School of Public Health and Preventative Medicine, Monash University, The Alfred Hospital, Melbourne, VIC 3004 Australia; 7 Department of Radiology, St Vincent’s Hospital, 390 Victoria Street, Darlinghurst, NSW 2010 Australia

**Keywords:** Allogeneic stem cells, Intra-articular injection, Knee function, Knee osteoarthritis, Knee pain, Mesenchymal stem cells, Magnetic resonance imaging, Visual analogue scale, WOMAC

## Abstract

**Background:**

Cell therapies are being investigated as potential disease modifying treatment options for osteoarthritis (OA). Progenza (PRG) comprises in vitro expanded mesenchymal stem cells derived from human donor adipose tissue combined with cell culture supernatant. The primary objective of this first-in-human study was to evaluate the safety and tolerability of PRG.

**Methods:**

We conducted a single centre, randomized, double-blind, placebo-controlled, single ascending dose study. Twenty patients aged 40–65 years with symptomatic Kellgren–Lawrence grade 1–3 knee OA were treated in two cohorts and randomized 4:1 to PRG or placebo. Cohort 1: 3.9 million cells (PRG 3.9M, n = 8) or placebo (n = 2) and cohort 2: 6.7 million cells (PRG 6.7M, n = 8) or placebo (n = 2). Each patient received a single intra-articular injection and was followed-up for 12 months.

**Results:**

The study population comprised 20 patients (placebo, n = 4; PRG 3.9M, n = 8; PRG 6.7M, n = 8). All patients reported at least one treatment-emergent adverse event (TEAE). The majority of events [143/169 (84.6%)] were mild with 34 (20.1%) being considered by the investigator to be treatment related. There were no serious AEs or withdrawals due to AEs during the study. There was a statistically significant within group improvement in VAS pain scores from baseline at all timepoints for the PRG combined group, with highly significant improvements seen at months 3, 6, 9 and 12 (p ≤ 0.005) while VAS pain scores in the placebo group showed marginal improvement. A statistically significant improvement was also seen in WOMAC pain subscale scores from baseline at all timepoints for the PRG combined group while a marginal improvement in the placebo group was not statistically significant. Between screening and month 12, there was no decrease in average lateral tibial cartilage volume in the PRG 3.9M group while the placebo group showed a statistically significant cartilage loss. This difference between the placebo and PRG 3.9M group was statistically significant (LSM difference 106.47 mm^3^, 95% CI 13.56 mm^3^, 199.37 mm^3^, p = 0.028).

**Conclusion:**

When administered as a single intra-articular injection to patients with symptomatic knee OA, PRG was safe and well tolerated. Furthermore, measurable improvements in symptoms and knee structure outcomes warrant further studies on PRG’s potential for disease modification in OA.

*Trial registration* ANZCTR, ACTRN12615000439549. Date registered: 7th May 2015, https://www.anzctr.org.au/Trial/Registration/TrialReview.aspx?id=368355

**Electronic supplementary material:**

The online version of this article (10.1186/s12967-018-1420-z) contains supplementary material, which is available to authorized users.

## Background

Osteoarthritis (OA) is a degenerative joint disease, mainly affecting weight-bearing joints such as hips, knees and ankles. Globally, OA is a major public health problem [1] and is the most common form of arthritis in Australia [2]. Self-reported data estimates that in 2014–2015, 2.1 million Australians (approximately 9% of the population) have OA; prevalence increases with age and it affects more females than males (10% versus 6%) [2].

OA is characterized by a progressive loss of articular cartilage, subchondral bone oedema, sclerosis, synovitis and marginal osteophyte formation. The main symptoms are pain, stiffness and limitation of joint movement. The symptoms and their severity vary by individual, but the condition gradually worsens over time and often results in significant functional impairment and reduced quality of life [3]. Although OA does not significantly impact mortality, it causes significant pain and disability, and is ranked 13th highest in global causes of years lived with disability [4].

There is no cure or disease-modifying treatment available for OA, with end stage symptomatic OA treated with costly joint replacement (arthroplasty). Current treatment modalities are classified as being either non-pharmacological, pharmacological and surgical [5]. Symptomatic relief is most often sought by physiotherapy, and exercise, topical applications, weight loss, dietary supplements, analgesics, corticosteroids or non-steroidal anti-inflammatory drugs (NSAIDs) [6]. More recently, injectable options such as hyaluronic acid (HA) and platelet rich plasma have also been used [7]. These applications, however, are associated with high outcome variability and, particularly with NSAIDs, associated with a high burden of iatrogenic events [8]. They are not effective in halting disease progression, and continued joint degeneration will eventually lead to joint replacement surgery [5]. Due to the limited lifespan of prostheses, particularly for the knee joint, along with inherent difficulties with revision surgeries, early joint replacement is relatively contraindicated.

Currently, cell therapies are being investigated as potential disease modifying treatment options for OA patients [7]. This includes both autologous and allogeneic mesenchymal stem cells (MSCs) derived from adipose tissue and bone marrow. MSCs are known to secrete a wide array of bioactive factors that modulate the inflammatory environment in an OA joint to a more anti-inflammatory environment, which promotes repair and regeneration. Whilst MSCs have the capacity to form de novo cartilage-like and bone-like cells in vitro [9], rather than participating directly through engraftment and cellular differentiation, their beneficial effects in OA are thought to be due to their immunomodulatory/anti-inflammatory properties coupled with their ability to prevent the dedifferentiation of chondrocytes into fibroblasts and stimulate chondrocyte type II collagen production [10].

Progenza (PRG) is composed of in vitro expanded MSCs derived from human donor adipose tissue combined with cell culture supernatant, frozen in vials ready to inject. MSCs are known to be immune privileged, enabling the administration of allogeneic cells of an human leukocyte antigen-unmatched donor to a patient without the need for concurrent immunosuppressive therapy [11]. The STEP (Safety, Tolerability and Efficacy of Progenza) Trial was a first-in-human study designed to further supplement these data by assessing the safety and preliminary efficacy of PRG in adults with knee OA. Its primary objective was to evaluate the safety and tolerability of PRG administered via a single intra-articular injection. Secondary objectives were to investigate the effect of PRG on pain, function and joint structures in the study knee, quality of life, and relevant serum and urine biomarkers.

## Methods

### Study design, participants and randomization

The STEP Trial was a randomized, double-blind, single centre, placebo-controlled, single ascending dose Phase I study. The trial was conducted between April 2015 and March 2017 at the Sydney Sportsmed Specialists and Sydney Sports Medicine Centre, Sydney, Australia. Investigational product (IP) administration was performed at East Sydney Private Hospital, Sydney, Australia and magnetic resonance imaging (MRI) was performed at Castlereagh Imaging, Sydney, Australia. Bellberry Ltd, Eastwood, Adelaide, Australia, granted ethical approval. The trial was prospectively registered with the Australian New Zealand Clinical Trials Registry (ACTRN12615000439549) and was performed with Good Clinical Practice in accordance with the requirements for the conduct of clinical studies set by the Clinical Trial Notification scheme of the Australian Therapeutic Goods Administration (TGA) and the Declaration of Helsinki. Written informed consent was obtained from all participants before entering the study.

Eligible patients were 40–65 years with a body mass index (BMI) of 20–30 kg/m^2^ inclusive and diagnosed Kellgren–Lawrence (KL) grade 1, 2 or 3 knee OA with moderate–severe pain in the study knee [35–90 mm on a 100 mm visual analogue scale (VAS)] at screening. Eligible patients were required to meet all inclusion criteria and were ineligible if they met any of the exclusion criteria (Table 1).

**Table 1:** Subject eligibility criteria

Inclusion criteria	Exclusion criteria
Provide written informed consent Males or females aged 40–65 years, inclusive Diagnosed KL grade 1, 2 or 3 knee OA in the study knee Moderate-severe pain associated with OA in the study knee as measured by a VAS pain score of between 35 and 90 mm inclusive at the screening visit BMI of 20–30 inclusive Negative results for virus antibody tests from samples taken at the screening visit: HIV 1 and 2 antibody test HCV antibody test HBV antibody test Able to read and write in English A female patient is eligible to enter the study if she meets following criteria: Not pregnant or breast feeding/lactating Females of non-childbearing potential (i.e., women who had a hysterectomy, had both ovaries surgically removed or have current documentation of tubal ligation, or are postmenopausal which is defined as 1 year without menses) Females of childbearing potential must agree to use adequate and highly effective methods of contraception throughout the study Male patients with female partners of childbearing potential must use adequate and highly effective methods of contraception such as double-barrier form for the entire duration of the study	Inability or unwillingness to comply with protocol requirements Evidence, or diagnosis, of OA in the non-study knee that is of a worse screening visit VAS score than the study knee Joint surgery in the study knee, including arthroscopy, within the last 3 years Consistently occurring major mechanical issues in the study knee including locking, catching and giving way Intra-articular injections into either knee within the last 3 months Current evidence of infection in either knee Diagnosed or symptomatic OA in other major joints (feet, hips, shoulders or spine) that is of greater clinical significance than the study knee Planned hip, knee, ankle or foot surgery including joint replacement within the expected study duration History or current evidence of other joint diseases (such as gout, rheumatoid arthritis and ankylosing spondylitis), or disease or medication affecting the bone or cartilage metabolism, including systemic corticosteroids and osteoporosis medication Unable to undergo an MRI scan for any reason including severe claustrophobia and metal implants such as hip, knee or aortic valve prosthetics Current smoker, or have been a regular (daily) smoker in the past 3 months Planned or current participation in any other interventional clinical trials Patients who require use of systemic immunosuppressants Any clinically significant condition(s) that in the opinion of the PI may compromise safety or compliance, interfere with evaluation or preclude completion of the study

BMI, body mass index; HBV, hepatitis B virus; HCV, hepatitis C virus; HIV, human immunodeficiency virus; KL, Kellgren–Lawrence; MRI, magnetic resonance imaging; OA, osteoarthritis; PI, principal investigator; VAS, visual analogue scale

Patients were treated in two cohorts of 10 patients each: cohort 1 received 3.9 million cells (PRG 3.9M) or placebo (cell culture media and cryopreservative) and cohort 2 received 6.7 million cells (PRG 6.7M) or placebo. A sequential group two-cohort design was used to first assess the blinded safety of the lower dose (PRG 3.9M) before enrolling the second cohort to receive a higher dose (PRG 6.7M). A statistician not directly involved in the conduct of the study prepared the randomization schedule using a block method. In each cohort, patients were randomized 4:1 via a secure customized central website to receive PRG or placebo, respectively. As this was a first-in-human study, a sentinel patient was included and the randomization was forced so the sentinel patient received a single-blind injection of PRG 3.9M. Allocation of all other patients to PRG (3.9M or 6.7M) or placebo occurred according to the randomization schedule. The participants, investigators, study coordinator and study team remained blinded to the treatment allocation throughout the trial.

The MSCs used for this study were derived from a single human donor, who was qualified according to TGA requirements [12]. Cells were isolated and culture-expanded in a good manufacturing practice accredited facility. The IP, 2 mL of either PRG 3.9M, PRG 6.7M or placebo, was provided by Regeneus Ltd (Ground Floor, 25 Bridge St, Pymble, NSW 2073, Australia) and stored in a CryoVial^®^ and maintained at or below − 150 °C prior to administration. The IP was thawed prior to being drawn up into a sterile syringe and administered via ultrasound guided intra-articular injection into the study knee either by an independent, unblinded radiologist or sports and exercise medicine physician trained in this technique. A screen was used to ensure the patient remained blinded to the treatment allocation.

Post-IP administration, patients were assessed at clinic visits on days 7 and 28, and months 3, 6, 9 and 12 to evaluate ongoing safety and efficacy. The Study Safety and Oversight Committee (SSOC) reviewed the safety data [comprising adverse events (AEs), physical examination findings, vital signs, electrocardiogram (ECG) results, medication usage, clinical chemistry and haematology results] from the sentinel patient following the day 7 visit and prior to enrolling the rest of cohort 1. Progression to cohort 2 was determined by the SSOC review of cohort 1 safety data comprising cumulative data up to the last patient’s day 28 visit.

### Safety and efficacy assessments

Safety assessments involved AE monitoring, vital signs, clinical laboratory parameters, physical examinations, ECG and documentation of concomitant medication use. AEs and concomitant medication use were collected at every visit and other safety measures were collected at pre-specified times throughout the study. Clinical chemistry and haematology analyses included liver and renal functions and full blood counts, respectively.

Patients completed self-reported pain, function and quality of life questionnaires [VAS, Western Ontario McMaster Universities Arthritis Index (WOMAC) LK3.1 and assessment of quality of life 4D questionnaire (AQoL-4D)]. The screening VAS result and day 1 (prior to IP administration) WOMAC and AQoL-4D results were used as a baseline for each patient. All three questionnaires were repeated at day 28 and months 3, 6, 9 and 12. Blood and urine samples were collected at all visits for biomarker analysis [urine: type II collagen C2C peptide (C2C) and C-terminal telopeptide of type II collagen (CTX-II); serum: macrophage migration inhibitory factor (MIF), HA and C-terminal telopeptide of type I collagen (CTX-I)]. Activity levels were measured in a sub-set of patients who provided consent to wear a FitBit^®^ Charge HR (FitBit, CA, USA) device on their wrist for the 7 days prior to each clinic visit.

### Imaging

At screening, patients underwent standard weight bearing bilateral knee X-rays, captured parallel to the tibial plateau in the Rosenberg view. An independent radiologist reviewed the X-rays and assigned a KL OA grading to each knee to determine study eligibility.

Knees were imaged on a 3T whole body magnetic resonance unit (Siemens Healthcare) prior to the treatment day and at month 12 visit. A T1-weighted coronal spin echo sequence was obtained as well as a 3D dual echo in steady state sequence in the double oblique sagittal orientation. MRIs were read blinded to treatment allocation but without blinding to the acquisition timepoint. Semi-quantitative scoring was performed according to the MRI OA Knee Score (MOAKS) system described in Hunter et al. [13].

Quantitative measurements of cartilage volume and bone area and additional semi-quantitative assessments of cartilage defects and bone marrow lesions (BML) were performed using validated methods as described by Wang et al. [14]. The coefficients of variation (CVs) for medial and lateral tibial and patellar cartilage volume measures were 3.4, 2.0 and 2.1% respectively [15, 16]. Medial and lateral tibial plateau bone areas were used as a measure of tibial bone size; CVs for the medial and lateral tibial plateau areas were 2.3 and 2.4% respectively [17]. Semi-quantitative measures were obtained using a modified International Cartilage Repair Society classification system [18]. A cartilage defect was included if present in at least two consecutive slices. A prevalent cartilage defect was defined as a cartilage defect score of ≥ 2 at any site within that compartment. Intra-observer reliability and inter-observer reliability (expressed as intraclass correlation coefficient) were 0.90 and 0.90 for the medial tibiofemoral compartment, and 0.89 and 0.85 for the lateral tibiofemoral compartment respectively [18]. BMLs were defined as areas of increased signal intensity within the subchondral bone region in either the distal femur, the proximal tibia, or patella [19]. A BML was identified as being definitely present if it appeared on two or more adjacent slices and was further classified as “small” (grade 1) or “large” (grade 2) as defined by Felson et al. [19]. The intra-observer reproducibility for determination of the BML was assessed using 60 randomly selected knee MRIs (κ value 0.88, p < 0.001).

### Statistical analysis

This was a first-in-human study with a primary objective of safety and tolerability; no formal sample size calculation was performed but available regulatory and industry guidance for first-in-human studies was referenced. All patients recruited to the study who received study treatment were included in the intent-to-treat (ITT) population for efficacy analyses and all patients in the ITT population who had at least one post-baseline safety assessment were included in the safety set for safety analyses. For analysis purposes, placebo patients from each cohort were pooled to produce a single placebo group.

Safety data and secondary efficacy results were summarized using descriptive statistics. A general repeated covariance pattern model was fitted to explore the difference between placebo and PRG in change from baseline in patient reported outcomes and imaging assessments for the ITT population. Patient was included as a random term to take into account the repeated measures nature of the data; timepoint, treatment group and baseline values were included as covariates. Change from baseline was presented as least squares mean estimates with 95% confidence intervals (CI), statistical significance was determined by a p value of < 0.05. All measures were analyzed using SAS software (V9.4, SAS Statistical Institute, Cart, NC, USA).

## Results

### Baseline characteristics and study participants

**Fig. 1:**
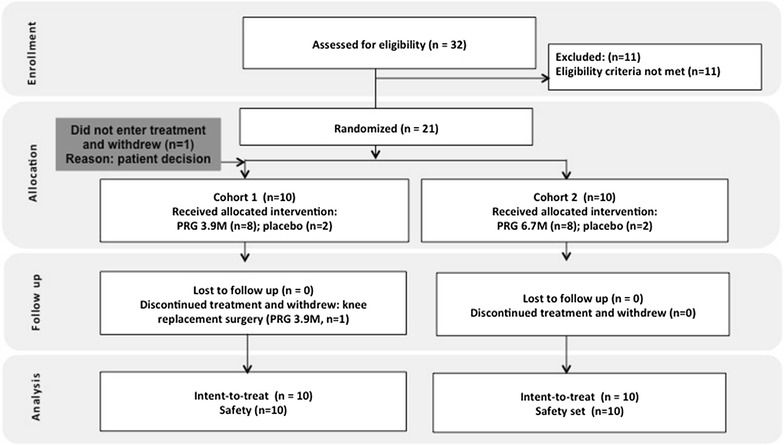
Study CONSORT flow diagram

From a total of 32 patients, 21 were eligible for randomization following screening (Fig. 1). One patient in cohort 1 withdrew (patient decision) prior to IP administration and was replaced, per the protocol; the remaining 20 patients that received treatment formed the ITT and safety sets. One PRG-treated patient in cohort 1 withdrew from the study after month 3, to proceed straight to total knee arthroplasty without obtaining other treatment options. There was excellent compliance with study assessments; only one visit was missed (PRG 3.9M group, month 6). A sub-set of 18 patients contributed FitBit^®^ data [4 (100%) placebo patients, 7 (87.5%) PRG 3.9M patients and 7 (87.5%) PRG 6.7M patients].

Baseline characteristics were generally similar between the groups (Table 2); however the placebo group had a higher proportion of females (75%) compared to the PRG groups (PRG 3.9M, 25%; PRG 6.7M 37.5%). The study population had a mean age of 53.3 ± 7.15 years. Most patients (80%) had bilateral knee OA with 75% in each group having KL grade 3 OA in their study knee.

**Table 2:** Summary of demography and baseline characteristics

	Placebo (n = 4)	PRG 3.9M (n = 8)	PRG 6.7M (n = 8)
Demographics			
Age (years)	55.0 ± 10.42	50.8 ± 7.29	55.0 ± 5.15
Females	3 (75%)	2 (25%)	3 (37.5%)
Height (cm)	165.0 ± 7.87	172.6 ± 10.99	174.4 ± 11.99
Weight (kg)	69.8 ± 11.55	82.9 ± 12.44	81.9 ± 14.23
BMI (kg/m^2^)	25.5 ± 2.84	27.7 ± 2.05	26.8 ± 2.98
OA characteristics			
Study knee KL OA grade 1	1 (25%)	0 (0%)	1 (12.5%)
Study knee KL OA grade 2	0 (0%)	2 (25%)	1 (12.5%)
Study knee KL OA grade 3	3 (75%)	6 (75%)	6 (75%)
OA in non-study knee	3 (75%)	7 (87.5%)	6 (75%)
Patient-reported outcomes			
VAS pain score (0–100 mm)	43.8 ± 7.41	57.0 ± 13.82	60.8 ± 13.01
WOMAC pain score (0–20)	6.3 ± 3.86	6.6 ± 2.07	7.9 ± 3.04
WOMAC stiffness score (0–8)	3.3 ± 2.06	3.4 ± 1.19	4.1 ± 1.89
WOMAC physical functioning score (0–68)	16.7 ± 10.69	22.0 ± 9.80	26.8 ± 10.20
AQoL-4D utility score	0.75 ± 0.213	0.80 ± 0.140	0.76 ± 0.183
Activity level (FitBit^®^; n = 18)			
Average daily steps	11,071 ± 7085	9049 ± 2605	11,934 ± 12,013
Quantitative MRI assessments			
Cartilage volume (mm^3^)			
Medial tibial region	1597.1 ± 642.95	2037.0 ± 665.59	2166.0 ± 858.29
Lateral tibial region	1777.5 ± 532.24	2459.3 ± 836.59	2470.3 ± 784.52
Patella	2588.3 ± 965.59	2895.2 ± 1204.61	3637.1 ± 1431.01
Tibial bone area (mm^2^)			
Medial region	2045.9 ± 374.36	2567.4 ± 388.86	2727.2 ± 632.68
Lateral region	1508.3 ± 364.83	1641.3 ± 368.14	1611.9 ± 582.25
Bone marrow lesions			
Medial tibiofemoral region	1 (25%)	4 (50%)	8 (100%)
Lateral tibiofemoral region	1 (33.3%)	4 (50%)	2 (25%)
Patella	2 (50%)	3 (37.5%)	1 (12.5%)
Cartilage defects			
Medial tibiofemoral region	3 (75%)	8 (100%)	7 (100%)*
Lateral tibiofemoral region	4 (100%)	8 (100%)	7 (100%)*
Patella	3 (75%)	6 (75%)	6 (85.7%)
Biomarkers			
Urine CTX-II (ng/mmol)	336.8 ± 311.34	149.9 ± 44.45	230.9 ± 136.69
Urine C2C (ng/mmol)	1591.6 ± 715.73	2388.9 ± 1616.07	1049.0 ± 1026.84
Serum HA (ng/mL)	32.6 ± 6.32	49.7 ± 19.95	47.0 ± 24.05
Serum MIF (ng/mL)	12.9 ± 3.32	13.2 ± 5.34	15.5 ± 2.86
Serum CTX-I (ng/mL)	0.3 ± 0.08	0.3 ± 0.15	0.4 ± 0.10

Data are presented as the mean ± SD or n (%). Baseline data from semi-quantitative analysis of MRI scans, conducted using the MRI osteoarthritis knee score (MOAKS) methodology are provided in Additional file 1: Table S1

AQoL-4D, assessment of quality of life 4D questionnaire; BMI, body mass index; C2C, type II collagen C2C peptide; CTX-I, C-terminal telopeptide of type I collagen; CTX-II, C-terminal telopeptide of type II collagen; HA, hyaluronic acid; KL, Kellgren–Lawrence; MIF, macrophage migration inhibitory factor; VAS, visual analogue scale; WOMAC Western Ontario McMaster Universities Arthritis Index

* Missing double echo steady state (DESS) sequence in 1 patient

### Adverse events

All patients experienced at least one treatment emergent adverse event (TEAE) during the 12-month course of the study (Table 3) with the majority of TEAEs being mild [143 (84.6%)] and unrelated to IP [135 (79.9%)]. The majority of patients experienced arthralgia [placebo: 4 (100%), PRG 3.9M: 6 (75%) and PRG 6.7M: 8 (100%)]. Approximately half of all patients experienced joint effusion [placebo: 3 (75%), PRG 3.9M: 6 (75%) and PRG 6.7M: 3 (37.5%)].

**Table 3:** Summary of treatment-emergent adverse events (TEAEs)

	Placebo (n = 4)	PRG 3.9M (n = 8)	PRG 6.7M (n = 8)
Patient summary			
TEAEs	4 (100.0)	8 (100.0)	8 (100.0)
Most common TEAEs^a^			
Arthralgia	4 (100.0)	6 (75.0)	8 (100.0)
Joint effusion	3 (75.0)	6 (75.0)	3 (37.5)
Upper respiratory tract infection	1 (25.0)	2 (25.0)	3 (37.5)
Joint stiffness	3 (75.0)	2 (25.0)	1 (12.5)
Joint lock	1 (25.0)	2 (25.0)	1 (12.5)
IP-related TEAEs	3 (75.0)	7 (87.5)	6 (75.0)
Arthralgia	3 (75.0)	4 (50.0)	5 (62.5)
Joint effusion	1 (25.0)	3 (37.5)	2 (25.0)
Joint stiffness	1 (25.0)	2 (25.0)	1 (12.5)
Bursitis	–	1 (12.5)	1 (12.5)
Joint swelling	–	1 (12.5)	1 (12.5)
Injection site pain	–	–	1 (12.5)
Joint lock	–	1 (12.5)	–
Joint warmth	–	1 (12.5)	–
Malaise	–	1 (12.5)	–
Paraesthesia	–	1 (12.5)	–
Event summary			
TEAEs	43	55	71
Mild	35 (81.4)	45 (81.8)	63 (88.7)
Moderate	8 (18.6)	9 (16.4)	8 (11.3)
Severe	0 (0.0)	1 (1.8)	0 (0.0)
SAEs	0 (0.0)	0 (0.0)	0 (0.0)
IP-related TEAEs	5 (11.6)	16 (29.1)	13 (18.3)

Data are presented as n (%) where n represents the number of patients or events

TEAE, treatment emergent adverse event; SAE, serious adverse event. IP-related events were AEs deemed by a blinded study investigator to be possibly, probably or definitely related to the study drug

^a^TEAEs occurring in > 4 patients across the trial

Most patients [16 (80%)] experienced at least one TEAE that was considered by the investigator to be IP related (Table 3), with a higher incidence of IP related events in the PRG groups [PRG 3.9M: 16/55 (29.1%); PRG 6.7M: 13/71 (18.3%)] compared to placebo [5/43 (11.6%)]. Arthralgia was the most common IP-related TEAE [placebo: 3 (75%), PRG 3.9M: 4 (50%) and PRG 6.7M: 5 (62.5%)] and was predominantly mild.

No serious AEs were reported and no patients withdrew from the study due to an AE. One severe AE of prepatellar bursitis (PRG 3.9M group) occurred 13 days after IP administration and was considered by the investigtor as possibly related to the intra-articular injection technique. A clear straw-coloured fluid (17 mL) aspirated from the bursa showed no growth on microbiology testing, and the condition resolved with treatment within 2 weeks. Moderate supra-patella bursitis, possibly related to the IP injection technique, occurred 2 weeks after administration of the IP in a patient in the PRG 6.7M group.

### Other safety assessments

The majority of clinical chemistry and haematology assessments were within the laboratory reference range in the majority of patients throughout the study. Clinically significant out of range results were reported in three patients (Table 4). Vital sign monitoring, urinalysis, complete and symptom-directed physical examinations and ECG evaluation did not reveal any significant abnormalities or patterns of concern.

**Table 4:** Summary of abnormal clinically significant haematology and clinical chemistry results

Group	Parameter	Visit	Value	Laboratory reference range (low, high)	Status
Placebo	Neutrophils	Month 12	1.6 10^9^/L	(2, 7.5)	L
	WBC	Month 12	3.6 10^9^/L	(4, 11)	L
PRG 6.7M	ALT	Month 12	69 U/L	(5, 40)	H
	AST	Month 12	96 U/L	(10, 40)	H
PRG 6.7M	Potassium	Month 6	5.6 mmol/L	(3.5, 5.5)	H

H, higher than laboratory reference range; L, lower than laboratory reference range; ALT, alanine aminotransferase; AST, aspartate aminotransferase; WBC, white blood cells

### Concomitant medications

The majority of patients [19 (95%)] reported using some form of analgesia or anti-inflammatory medicine prior to study commencement. Several patients received short courses of additional analgesia during the study; however, in the majority of cases these were for indications unrelated to pain in their study knee. No trends were noted regarding the use of concomitant analgesia or anti-inflammatory medicines during the study and there were no differences across treatment groups.

### Effect of PRG on knee pain and function

**Fig. 2:**
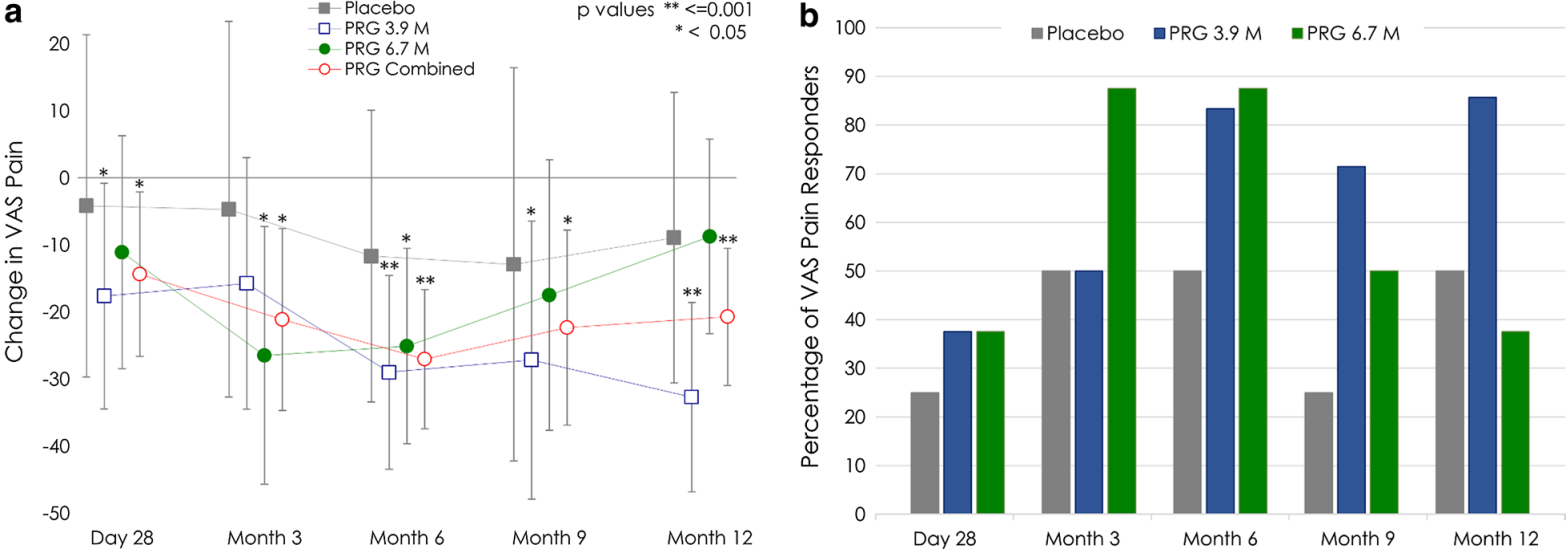
**a** Change from baseline VAS pain scores (data are presented as the least squares mean estimates with 95% confidence intervals and within group p values) and **b** proportion of pain responders (responders with an improvmeent of at least 30% from baseline VAS score). VAS, visual analogue scale

There was a statistically significant within group improvement in VAS pain scores from baseline at all timepoints for the PRG combined group (Fig. 2a) with highly significant improvements observed from months 3, 6, 9 and 12 (p ≤ 0.005). The reduction in VAS pain scores was maintained to month 12 for the PRG 3.9M group (− 32.7 mm, 95% CI − 46.83, − 18.56 mm, p < 0.001). The largest reduction in VAS pain for the PRG 6.7M group was seen at month 3 (− 26.46 mm, 95% CI − 45.69, − 7.22 mm, p = 0.01). The VAS pain scores in the placebo group showed some improvement (Fig. 2a), these were not statistically significant.

The overall average change in VAS pain scores across all timepoints showed a statistically significant within group reduction from baseline in both PRG groups (PRG 3.9M p = 0.002; PRG 6.7M p = 0.018) and the PRG combined group (p < 0.001). There was a marginal decrease in mean VAS score in the placebo group (− 8.46 mm, 95% CI − 29.89, 12.96, p = 0.416).

Patients who reported at least a 30% improvement from baseline in VAS score were classified as pain responders. A maximum of 50% of placebo patients and 87.5% of PRG-treated patients were considered responders (Fig. 2b). The percentage of responders in the PRG-treated groups was higher than that in the placebo group at almost all timepoints measured. There was a wide spread in the VAS pain score data at all timepoints in the placebo group, largely driven by single patient who had a very substantial reduction in VAS pain score (placebo-responder).

**Fig. 3:**
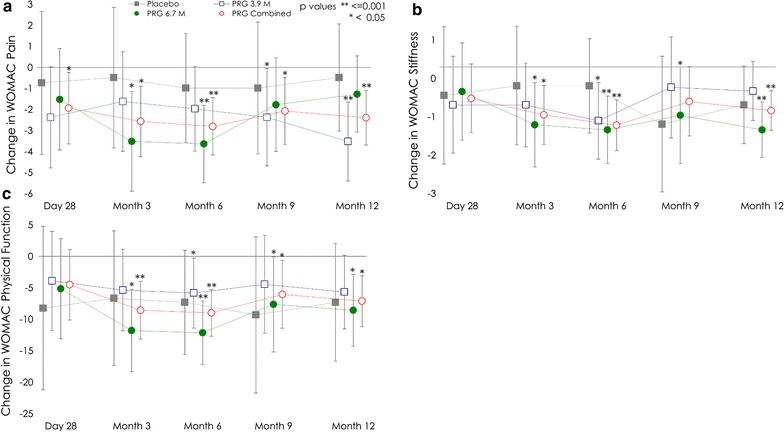
Change from baseline in WOMAC **a** pain subscale scores, **b** stiffness subscale scores and **c** physical function subscale scores. Data are presented as the least squares mean estimates with 95% confidence intervals and within group p values. WOMAC Western Ontario McMaster Universities Arthritis Index

There was a statistically significant within group improvement in WOMAC pain subscale scores from baseline at all timepoints for the PRG combined group (Fig. 3a) with improvements observed from months 3, 6, 9 and 12 (p ≤ 0.014). The WOMAC pain subscale scores showed marginal improvement in the placebo group. Analysis of WOMAC pain subscale scores across all timepoints showed a statistically significant reduction from baseline in both PRG dose groups (PRG 3.9M − 2.37, 95% CI − 4.08, − 0.66, p = 0.010 and PRG 6.7M: − 2.34, 95% CI − 4.06, − 0.63, p = 0.011) and in the PRG combined group (− 2.35, 95% CI − 3.56, − 1.15, p < 0.001). Overall change in the placebo group was marginal and did not reach statistical significance (− 0.73, 95% CI − 3.14, 1.67, p = 0.526).

WOMAC stiffness and physical function subscale assessments for PRG combined showed statistically significant improvements at months 3, 6, 9 and 12, with the exception of month 9 stiffness. The placebo group also showed reduction in subscale scores at all timepoints but did not reach statistical significance (Fig. 3b, c). Similarly, analysis of overall average change across all timepoints showed statistically significant improvement from baseline in WOMAC stiffness and physical function subscales in both PRG dose groups, but not in the placebo group. There were no statistically significant differences at any timepoint between placebo and PRG groups for the WOMAC subscale scores.

### Effect of PRG on knee structure

Between screening and month 12, there was no decrease in average lateral tibial cartilage volume in the PRG 3.9M group [11.1 mm^3^ (95% CI − 37.1 mm^3^, 59.3 mm^3^, p = 0.628)] while the placebo group showed a statistically significant cartilage loss [− 95.4 mm^3^ (95% CI − 172.6 mm^3^, − 18.2 mm^3^, p = 0.019)]. The difference between the placebo and the PRG 3.9M group was statistically significant (least squares mean difference 106.5 mm^3^, 95% CI 13.6 mm^3^, 199.4 mm^3^, p = 0.028). A statistically significant within group decrease was also observed in the PRG 6.7M group (− 78.0 mm^3^, 95% CI − 125.9 mm^3^, − 30.1 mm^3^, p = 0.004).

The placebo group contained proportionally more females than did the PRG groups and, to account for lower baseline cartilage volume in female patients, percentage change analyses were undertaken. Similar to the improved finding in the PRG 3.9M group for cartilage volume, there was a significant difference in percent change of lateral tibial cartilage volume between the placebo and PRG 3.9M groups of 5.4% (95% CI 0.9%, 9.9%, p = 0.022). The statistically significant expansion in absolute medial tibial bone area from screening to month 12 for the PRG 3.9M group (50.3 mm^2^, 95% CI from 1.1 mm^2^, 99.6 mm^2^, p = 0.046) was not significant when analysed as percentage change (Table 5). There was no significant difference in tibial bone area change among the treatment groups.

**Table 5:** Quantitative MRI results: change from screening to month 12 in tibial cartilage volume and bone area

	Placebo (n = 4)	PRG 3.9M (n = 8)	PRG 6.7M (n = 8)	Placebo – PRG 3.9M	Placebo – PRG 6.7M
Lateral tibial cartilage volume, mm^3^	− 95.4 (− 172.6, − 18.2) p = 0.019	11.1 (− 37.1, 59.3) p = 0.628	− 78.0 (− 125.9, − 30.1) p = 0.004	106.5 (13.6, 199.4) p = 0.028	17.4 (− 74.6, 109.4) p = 0.690
Lateral tibial cartilage volume, % change	− 5.0 (− 8.8, − 1.3) p = 0.012	0.4 (− 2.0, 2.7) p = 0.730	− 3.5 (− 5.8, − 1.2) p = 0.006	5.4 (0.9, 9.9) p = 0.022	1.5 (− 2.9, 6.0) p = 0.475
Medial tibial cartilage volume, mm^3^	− 15.4 (− 166.8, − 136.0) p = 0.830	− 30.3 (− 141.2, 80.5) p = 0.567	− 73.8 (− 186.1, 38.6) p = 0.181	− 14.9 (− 203.2, 173.4) p = 0.868	− 58.3 (− 250.6, 133.9) p = 0.526
Medial tibial cartilage volume, % change	− 1.7 (− 8.8, 5.3) p = 0.607	− 1.5 (− 6.7, 3.6) p = 0.532	− 3.5 (− 8.7, 1.8) p = 0.178	0.2 (− 8.6, 9.0) p = 0.964	− 1.7 (− 10.7, 7.2) p = 0.685
Lateral tibial bone area, mm^2^	− 10.0 (− 70.9, 50.9) p = 0.730	− 8.5 (− 54.4, 37.4) p = 0.698	25.6 (− 71.6, 20.4) p = 0.253	1.5 (− 74.7, 77.7) p = 0.967	− 15.6 (− 92.1, 60.9) p = 0.669
Lateral tibial bone area, % change	− 0.2 (− 4.0, 3.6) p = 0.906	− 0.2 (− 3.1, 2.7) p = 0.888	− 2.0 (− 4.9, 0.9) p = 0.158	0.0 (− 4.8, 4.8) p = 0.993	− 1.8 (− 6.6, 3.0) p = 0.436
Medial tibial bone area, mm^2^	30.4 (− 41.6, 102.4) p = 0.381	50.3 (1.1, 99.6) p = 0.046	− 17.0 (− 68.1, 34.1) p = 0.487	20.0 (− 68.6, 108.5) p = 0.636	− 47.4 (− 140.4, 45.6) p = 0.293
Medial tibial bone area, % change	1.4 (− 1.6, 4.3) p = 0.336	2.0 (− 0.0, 4.0) p = 0.051	− 1.0 (− 3.1, 1.1) p = 0.326	0.6 (− 3.0, 4.3) p = 0.712	− 2.4 (− 6.2, 1.4) p = 0.205

Increase in cartilage volume = improvement; Increase in bone area = worsening

Other quantitative MRI measurements (BMLs and cartilage defects) were similar between the groups at screening (Table 2) and there were very few changes in these parameters over the course of the trial. The majority of patients (> 75%) had a cartilage defect in the medial tibiofemoral, lateral tibiofemoral and/or patella regions at baseline. All patients in the PRG 6.7M group had a BML in the medial tibiofemoral regions, versus 50% in the PRG 3.9M group and 25% in the placebo group. The only changes from screening were a reduction in patella BMLs at month 12 in a patient in the PRG 3.9M group and an increase in tibiofemoral BMLs at month 12 in three patients (PRG 3.9M group 1 patient; PRG 6.7M group 2 patients). MOAKS derived measures were similar between the groups at screening and the majority remained unchanged from screening to month 12 (Additional file 1: Tables S1, S2).

### Other efficacy outcomes

The FitBit^®^ data showed average activity levels remained consistently around 10,000 steps per day in each group, indicating no substantial change in activity levels during the trial.

There were no statistically significant differences in the AQoL-4D utility scores between placebo and the PRG groups at any timepoint. Within group longitudinal analysis showed statistically significant non-zero changes from baseline in the PRG 3.9M group at month 6 (0.073, 95% CI 0.000, 0.146, p = 0.050), and month 12 (0.074, 95% CI 0.004, 0.114, p = 0.039. In each instance, the direction of change was positive, indicating an improvement in overall utility score from baseline levels. There were marginal changes in the AQoL-4D utility score over time for both the placebo and PRG 6.7M groups, which were not statistically significant.

Overall there was little change in the mean biomarker levels over the course of the trial, most within and between group results were not statistically significant.

## Discussion

The STEP trial met its predefined endpoint, demonstrating safety and tolerability of PRG given as a single intra-articular injection at doses of 3.9M or 6.7M cells. All patients reported at least one AE, the majority of which were mild and considered unrelated to the IP. There were no AE-related withdrawals or serious AEs during the study. The incidence and nature of AEs was within expectations and is consistent with the findings of previous studies evaluating intra-articular injection of autologous adipose-derived MSCs and bone-marrow derived MSCs [20–23].

Some interesting preliminary efficacy results were obtained. There was a reduction in VAS pain scores and WOMAC pain subscale scores first seen at day 28 in both PRG dose groups, which was maintained over the course of the trial. These results are consistent with patient-reported outcomes in other studies investigating the use of cellular therapy for the treatment of OA-affected joints [21, 24–28]. However, most of these studies did not include a control or placebo group.

The effect of placebo on pain is well-documented in OA studies [29] and is enhanced with more invasive and more frequent interventions [30]. One placebo-treated patient in our trial reported a marked improvement in VAS pain score, whereas in the other placebo-treated patients VAS pain scores generally worsened. The 95% confidence intervals for the pooled placebo group were therefore very wide and it was not possible to reasonably see a statistically significant difference between the placebo and the PRG-treated groups.

The therapeutic effect of cell therapy, such as that described in this study, is likely to proceed in two phases. The first is the reduction in pain attributable to the capacity of cells to secrete bioactive factors. These factors are thought to modulate the environment in the joint from a pro-inflammatory state to a more anti-inflammatory state. Unlike other cell therapies, PRG contains cell culture supernatant that is rich in these bioactive factors. The reduction in pain observed in the PRG treated patients in this study is possibly attributable to the initial anti-inflammatory actions of these bioactive factors, and sustained by the continued secretion of these factors by the MSCs injected into the joint. The second, and longer-term, phase is the ability of MSCs to embed in the joint tissues and potentially stimulate the repair and regeneration of damaged tissues, including cartilage.

Lateral tibial cartilage loss was halted in the PRG 3.9M group (0.4%) as compared to the placebo group (− 5%), the latter is consistent with reported annual rates of lateral tibial cartilage loss (− 5.3%) in OA patients [15]. Although our trial comprises small patient numbers, the effect on the lateral tibial cartilage in the PRG 3.9M group concurs with pre-clinical study findings with PRG (manuscript in preparation). The PRG 6.7M group exhibited some lateral tibial cartilage loss at a slower rate (− 3.5%) than the placebo patients although it is unclear why the cartilage loss in the higher dose PRG group was greater than in the lower dose group. Taken together, the positive cartilage results from the pre-clinical and clinical studies suggest PRG may slow the progression of OA. Beneficial effects largely observed in the lateral tibial region has been a common finding in a number of studies [31, 32]. It has been speculated that although OA is a disease that affects the entire joint, in general the medial tibiofemoral region is more severely affected than the lateral tibiofemoral region. Therefore, there may be less opportunity to demonstrate improvement in the medial tibiofemoral region simply because it has later stage disease.

### Limitations

The major limitation of our study included its small size for efficacy endpoints; however it was an appropriate size for safety assessment in a first-in-human study.

In our study, baseline serum CTX-I, HA and urine C2C and CTX-II values were comparable to those published [33], however, there was little change in biomarkers over the course of the study. In future studies with PRG, biomarker analysis in larger populations may help to reduce inter-patient variations and reveal stronger associations with MRI results. Additionally, measuring these biomarkers in synovial fluid may contribute to a clearer understanding of the impact of cell therapy in this setting.

The use of the FitBit^®^ HR activity monitors in this study by consenting patients was exploratory. Whilst it was hoped that parameters such as minutes of activity, minutes asleep, calories burnt, floors climbed could be analysed, the data collected were generally unreliable. Thus analysis was limited only to the average number of steps taken.

Analgesics and NSAIDs are typical first-line treatments used for symptom relief in OA patients [34, 35]. Most patients reported using analgesia or anti-inflammatory medication prior to study commencement and were asked at every clinic visit to report any new or changed medications. However during the latter stages of the trial study visits were 3 months apart and patient recall was poor. A different strategy to capture medication use in future trials, such as a patient diary, may enable a thorough analysis.

Lastly, due to the small number of patients and the exploratory nature of the secondary efficacy assessments, no adjustments for multiplicity were employed for the statistical analysis. Larger trials powered for efficacy would be required to investigate and confirm the therapeutic benefits observed in this study.

## Conclusion

The results of the STEP trial support that, when administered as a single intra-articular injection to patients with symptomatic KL grade 1, 2 or 3 OA, PRG (3.9M dose and 6.7M dose) is both safe and well tolerated. Despite the small study size, within group improvements were seen across a number of efficacy measures in the PRG groups. Improvements in pain scores and quantitative MRI results were seen in the PRG groups. The findings observed in our study are encouraging and warrant additional trials to confirm the safety and further explore the potential for disease modifying effects of PRG in knee OA.

## Additional file

**Additional file 1: Table S1.** Baseline distribution of semi-quantitative MRI OA Knee Score (MOAKS) markers. **Table S2.** Number of patients noted with changes from screening to month 12 for MOAKS assessments.

## Abbreviations

AE: adverse event
AQoL-4D: assessment of quality of life 4D questionnaire
BMI: body mass index
BML: bone marrow lesion
C2C: type II collagen C2C peptide
CI: confidence interval
CTX-I: C-terminal telopeptide of type I collagen
CTX-II: C-terminal telopeptide of type II collagen
CVs: coefficients of variation
ECG: electrocardiogram
HA: hyaluronic acid
IA: intra-articular
IP: investigational product
ITT: intent to treat
KL: Kellgren–Lawrence
MIF: macrophage migration inhibitory factor
MOAKS: magnetic resonance imaging osteoarthritis knee score
MRI: magnetic resonance imaging
MSC: mesenchymal stem cell
NSAID: non-steroidal anti-inflammatory drug
OA: osteoarthritis
PRG: Progenza
SSOC: Study Safety and Oversight Committee
STEP: Safety, Tolerability and Efficacy of Progenza
TEAE: treatment emergent adverse event
TGA: Therapeutic Goods Administration
VAS: visual analogue scale
WOMAC: Western Ontario McMaster Universities Arthritis Index
