# Supplementary material for: Safety, tolerability and efficacy of intra-articular Progenza in knee osteoarthritis: a randomized double-blind placebo-controlled single ascending dose study
Source: J Transl Med. 2018 Mar 6;16:49. doi: 10.1186/s12967-018-1420-z (PMC5840781; doi:10.1186/s12967-018-1420-z)
Supplement: Supplementary file 1 — Additional file 1: Table S1. Baseline distribution of semi-quantitative MRI OA Knee Score (MOAKS) markers. Table S2. Number of patients noted with changes from screening to month 12 for MOAKS assessments. [file 12967_2018_1420_MOESM1_ESM.pdf]

Additional File

Safety, tolerability and efficacy of intra-articular Progenza in knee osteoarthritis: A randomized double-blind placebo-controlled single ascending dose study

Additional Table S1

Baseline distribution of semi-quantitative MRI OA Knee Score (MOAKS) markers.

	Placebo (n=4)	PRG 3.9M (n=8)	PRG 6.7M (n=8)
Cartilage morphology			
Maximum thickness score across entire knee			
0	1 (25%)	0 (0%)	0 (0%)
1	0 (0%)	1 (12.5%)	0 (0%)
2	1 (25%)	3 (37.5%)	3 (37.5%)
3	2 (50%)	4 (50%)	5 (62.5%)
No. of subregions with thickness score >0 across entire knee			
0	1 (25%)	0 (0%)	0 (0%)
1-3	1 (25%)	2 (25%)	3 (37.5%)
4-6	1 (25%)	4 (50%)	3 (37.5%)
7-9	1 (25%)	2 (25%)	2 (25%)
Maximum surface area score across entire knee			
0-1	1 (25%)	0 (0%)	0 (0%)
2	0 (0%)	2 (25%)	2 (25%)
3	3 (75%)	6 (75%)	6 (75%)
No. of subregions with surface area score >0 across entire knee			
0-1	1 (25%)	0 (0%)	0 (0%)
2-4	0 (0%)	1 (12.5%)	1 (12.5%)
5-7	0 (0%)	2 (25%)	4 (50%)
8+	3 (75%)	5 (62.5%)	3 (37.5%)
Osteophytes			
No. of locations affected by any osteophyte			
0-2	1 (25%)	1 (12.5%)	0 (0%)
3-5	0 (0%)	0 (0%)	1 (12.5%)
6+	3 (75%)	7 (87.5%)	7 (87.5%)
Maximum osteophyte score in knee			
0-1	1 (25%)	1 (12.5%)	0 (0%)
2	1 (25%)	3 (37.5%)	6 (75%)
3	2 (50%)	4 (50%)	2 (25%)
Meniscus morphology			
Maximum grade across all locations			
0 (normal/signal)	1 (25%)	0 (0%)	0 (0%)
1 (tear)	0 (0%)	3 (37.5%)	3 (37.5%)
2 (maceration)	3 (75%)	5 (62.5%)	5 (62.5%)
BMLs			
No. of subregions affected by any BML			
0	1 (25%)	0 (0%)	0 (0%)
1	0 (0%)	2 (25%)	0 (0%)
2	0 (0%)	0 (0%)	0 (0%)
3	1 (25%)	1 (12.5%)	2 (25%)
4	0 (0%)	3 (37.5%)	1 (12.5%)
5+	2 (50%)	2 (25%)	5 (62.5%)
Maximum BML score in knee			
0	1 (25%)	0 (0%)	0 (0%)
1	0 (0%)	1 (12.5%)	0 (0%)
2	1 (25%)	5 (62.5%)	4 (50%)
3	2 (50%)	2 (25%)	4 (50%)
Synovitis (Hoffa-synovitis score)			
0	0 (0%)	0 (0%)	0 (0%)
1	2 (50%)	5 (62.5%)	3 (37.5%)
2-3	2 (50%)	3 (37.5%)	5 (62.5%)
Effusions (effusion-synovitis score)			
0-1	2 (50%)	1 (12.5%)	3 (37.5%)
2-3	2 (50%)	7 (87.5%)	5 (62.5%)

Data are presented as n (%). BML, bone marrow lesion.

Additional Table S2

Number of patients noted with changes from screening to Month 12 for MOAKS assessments.

Treatment group	MOAKS derived measure	Improved	Unchanged	Deteriorated
**Placebo**	Number of sub-regions with cartilage thickness score >0	0 (0%)	3 (75%)	1 (25%)
	Maximum BML score in knee	0 (0%)	3 (75%)	1 (25%)
	Number of sub-regions affected by BMLs	2 (50%)	2 (50%)	0 (0%)
	Hoffa-synovitis score	1 (25%)	3 (75%)	0 (0%)
	Effusion-synovitis score	2 (50%)	2 (50%)	0 (0%)
**PRG 3.9M**	Maximum cartilage thickness score across treated knee	0 (0%)	6 (86%)	1 (14%)
	Number of sub-regions with cartilage surface area score >0	0 (0%)	6 (86%)	1 (14%)
	Number of locations affected by osteophytes	0 (0%)	6 (86%)	1 (14%)
	Maximum BML score in knee	0 (0%)	5 (71%)	2 (29%)
	Number of sub-regions affected by BMLs	1 (14%)	4 (57%)	2 (29%)
	Hoffa-synovitis score	1 (14%)	6 (86%)	0 (0%)
	Effusion-synovitis score	0 (0%)	6 (86%)	1 (14%)
	Meniscus morphology – medial body sub-region grading	0 (0%)	6 (86%)	1 (14%)
	Meniscus morphology – maximum grade across all regions	0 (0%)	6 (86%)	1 (14%)
	Meniscus morphology – patient group	0 (0%)	6 (86%)	1 (14%)
**PRG 6.7M**	Maximum cartilage thickness score across treated knee	0 (0%)	7 (88%)	1 (13%)
	Number of sub-regions with cartilage surface area score >0	0 (0%)	7 (88%)	1 (13%)
	Maximum osteophyte score across treated knee	0 (0%)	7 (88%)	1 (13%)
	Number of locations affected by osteophytes	0 (0%)	7 (88%)	1 (13%)
	Maximum BML score in knee	1 (13%)	4 (50%)	3 (38%)
	Number of sub-regions affected by BMLs	1 (13%)	4 (50%)	3 (38%)
	Hoffa-synovitis score	0 (0%)	7 (88%)	1 (13%)
	Effusion-synovitis score	1 (13%)	7 (88%)	0 (0%)
	Meniscus morphology – lateral anterior sub-region grading	0 (0%)	7 (88%)	1 (13%)

Data are presented as n (%).BML, bone marrow lesion.
